# Postural Orthostatic Tachycardia Syndrome Associated with COVID-19: A Narrative Review

**DOI:** 10.3390/medicina60081325

**Published:** 2024-08-15

**Authors:** Jung-Hyun Park, Somin Park, Na-Hye Kim, Yoonjin Lee, Yoonkyung Chang, Tae-Jin Song

**Affiliations:** 1 Ewha Womans University College of Medicine, Seoul 07804, Republic of Korea; hyunn0o@ewhain.net (J.-H.P.); cottonseed@ewhain.net (S.P.); nh1115@ewhain.net (N.-H.K.); clover929@ewhain.net (Y.L.); 2 Department of Neurology, Mokdong Hospital, Ewha Womans University College of Medicine, Seoul 07985, Republic of Korea; ykchang@ewha.ac.kr; 3 Department of Neurology, Seoul Hospital, Ewha Womans University College of Medicine, Seoul 07804, Republic of Korea

**Keywords:** postural orthostatic tachycardia syndrome (POTS), COVID-19, renin–angiotensin–aldosterone system (RAAS) dysregulation, hyperadrenergic reaction, direct viral infection, tilt-up table test

## Abstract

Postural orthostatic tachycardia syndrome (POTS) is a complex condition marked by an atypical autonomic response to standing, leading to orthostatic intolerance and significant tachycardia without accompanying hypotension. In recent studies, a considerable number of individuals recovering from COVID-19 have been reported to experience POTS within 6 to 8 months post-infection. Key symptoms of POTS include fatigue, difficulty with orthostatic tolerance, tachycardia, and cognitive challenges. The underlying causes of POTS following COVID-19 remain unknown, with various theories proposed such as renin–angiotensin–aldosterone system (RAAS) dysregulation, hyperadrenergic reaction, and direct viral infection. Healthcare professionals should be vigilant for POTS in patients who have recovered from COVID-19 and are experiencing signs of autonomic dysfunction and use diagnostic procedures such as the tilt-up table test for confirmation. COVID-19-related POTS should be approached with a holistic strategy. Although many patients show improvement with initial non-drug treatments, for subjects who do not respond and exhibit more severe symptoms, medication-based therapies may be necessary. The current understanding of COVID-19-related POTS is limited, underscoring the need for more research to increase knowledge and enhance treatment approaches.

## 1. Introduction

The COVID-19 pandemic that emerged at the end of 2019 resulted in numerous adverse effects for millions of people worldwide, including death [1]. Patients with numerous associated diseases, including cardiac problems, insulin resistance, dementia, or epilepsy, have a bad prognosis after COVID-19 [2,3,4,5,6,7,8]. Recently, various sequelae, including those involving the respiratory and nervous systems, have been suggested to be associated with poor long-term prognosis after COVID-19 [9]. Among subjects affected by the COVID-19 pandemic and endemic, postural orthostatic tachycardia syndrome (POTS) has emerged as a major autonomic dysfunction syndrome [10,11].

### 1.1. Definition and Clinical Features of POTS

POTS is a continuous heart rate increase irrelevant to orthostatic hypotension. Specifically, POTS patients complain of postural symptoms of increased heart rate of more than 30 beats per minute for adults or at least 40 beats per minute or a maximum heart rate over 130 beats per minute for children aged 6–12 years, or >125 beats per minute for adolescents aged 13–18 years within 10 min of assuming an upright or standing position without another explainable etiology or orthostatic hypotension [11,12]. These clinical findings should be aligned with clinical symptoms indicative of POTS, persisting for a minimum of 6 months, before a definitive diagnosis of POTS can be established [13,14].

POTS is characterized by a wide range of chronic symptoms that are notably different from the related orthostatic changes. The symptoms usually appear in individuals between 15 and 50 years of age, with an average age of onset of approximately 30 years. A significantly higher (5-fold) occurrence is observed in females, particularly in those of childbearing age [12]. The level of functional disability experienced by young adults with POTS is comparable to that observed in conditions such as chronic obstructive pulmonary disease or congestive heart failure, frequently leading to a significantly reduced quality of life [12].

POTS symptoms can be divided into postural and non-postural symptoms. Reportedly, postural symptoms include vertigo, dizziness, palpitation, syncopal attack, weakness of extremities, tremor, dyspnea, and chest pain [15]. It is not uncommon for patients with POTS to experience episodes of fainting, although instances of presyncope are far more prevalent [16]. Approximately 10% of individuals with POTS report a family history of tachycardia or orthostatic intolerance [12]. The episodes are often triggered by factors such as postprandial status, physical activity, and heat. Other stressors such as trauma, electrical injuries, surgical procedures, pregnancy, autoimmune conditions, and significant psychosocial stress have been noted [17]. Symptoms often worsen during the perimenstrual period. The nature of the episodes associated with orthostatic position is typically cyclical, with variations including weight changes and fluctuations in blood pressure and heart rate [18,19].

Various non-postural symptoms can occur in patients with POTS. A significant portion of non-postural symptoms is gastrointestinal, including abdominal discomfort, nausea, vomiting, constipation, and diarrhea [19]. Patients with POTS tend to have a decrease in venous return due to the release of histamine or localized small molecules, which may cause vasodilation [19]. Furthermore, patients with irritable bowel syndrome frequently experience abdominal venous congestion or pooling, which may be why POTS patients frequently suffer from irritable bowel syndrome [20]. A delay in gastric transit time for accompanying autonomic dysfunction also leads to gastrointestinal disturbance in patients with POTS [21].

Neurologically, many patients with POTS experience cognitive impairments often described as “brain fog”, which includes challenges in thinking and concentrating, confusion, memory impairment, or a sensation of haziness or fuzziness in the head [22]. Dysfunction of the catecholamine pathway and its related brain structures might be the cause of this dysfunction [22]. Bladder dysfunction such as urinary frequency, incontinence, and urgency can occur due to autonomic dysfunction of the lower urinary tract system in patients with POTS [23]. Furthermore, sympathetic activation and cutaneous vasoconstriction might cause urticaria, Raynaud’s phenomenon, or livedo reticularis in POTS patients [24]. Additional common symptoms reported in POTS include migraines, anxiety, fatigue, unusual chest discomfort, sleep disturbances, and a reduced tolerance for physical exercise [11].

When standing from a normal lying posture, mean arterial blood pressure can decrease due to a reduction in venous return. When a decrease in blood pressure is detected by a baroceptor located at the carotid sinus and aortic arch, baroreceptor firing decreases, parasympathetic tone decreases, and sympathetic tone increases. Therefore, heart rate and rate of systemic vasoconstriction can increase. Rapid baroreflex control is also essential for the control of heart rate and peripheral vascular resistance during orthostatic stress. In the standing position, if the baroreflex does not function properly and deterioration of the baroreflex persists for an extended period despite reduced venous return, a continuous decrease in baroreceptor stretch can result. Consequently, exaggerated and persistent tachycardia can occur. These events are proposed as the main mechanisms of POTS [21].

Prior to diagnosis of POTS, patients often report illnesses involving viral infections in the upper respiratory or gastrointestinal systems. Identified viruses and bacteria associated with POTS include influenza, Epstein–Barr virus, and *Borrelia burgdorferi* [15]. In addition, POTS has been observed in conjunction with various autoimmune and connective tissue disorders, including Hashimoto’s thyroiditis, irritable bowel syndrome, rheumatoid arthritis, and Ehlers–Danlos syndrome [25].

### 1.2. Current Clinical Spectrum

**Figure 1:**
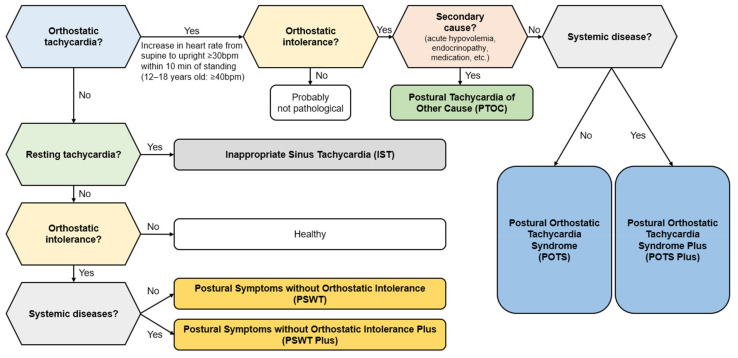
Algorithmic approach to postural orthostatic tachycardia syndrome (POTS) diagnosis [26].

The latest classification system proposed by the Canadian Cardiovascular Society in 2020 offers a more comprehensive view of the range of conditions associated with POTS (Figure 1) [26]. This new system introduces terms such as POTS Plus, Postural Symptoms without Orthostatic Intolerance (PSWT), PSWT Plus, and Postural Tachycardia of Other Cause (PTOC), expanding on the traditional definition of POTS. POTS Plus is characterized by the standard orthostatic symptoms in addition to a range of non-cardiovascular issues such as gastric emptying problems, intractable vomiting, severe constipation, neurogenic bladder, severe chronic pain, intractable headaches, significant flushing, anaphylaxis symptoms, and severe food intolerances. Associated conditions include hypermobile Ehlers–Danlos syndrome, hypermobile spectrum disorder, mast cell activation disorder, chronic fatigue syndrome/myalgic encephalomyelitis, Celiac disease, autoimmune disorder, chronic migraines, cerebrospinal fluid leak, mitochondrial mutations disorders, and multiple sclerosis. PSWT represents individuals who exhibit typical POTS orthostatic symptoms but do not fulfill the hemodynamic criteria for POTS. The PSWT Plus category is for patients with PSWT who also experience non-cardiovascular symptoms and comorbidities similar to those observed in POTS Plus. PTOC is used to classify patients who meet the hemodynamic criteria for POTS but whose tachycardia is caused by other identifiable factors such as anemia, anxiety, fluid loss, hormonal imbalances, side effects of medications, drug abuse, or extended periods of inactivity [26].

## 2. COVID-19-Related POTS

### 2.1. Definition

Determining the exact prevalence of COVID-19-related POTS is challenging because it is difficult to provide evidence that POTS occurred due to or related to COVID-19. However, in 2021, the American Autonomic Society released a statement defining long COVID POTS, highlighting the range of symptoms observed following COVID-19. These symptoms include palpitation, shortness of breath, fatigue, chest pain, cognitive dysfunction, sleep problems, difficulty standing due to orthostatic intolerance, abdominal pain, neuropathic symptoms, gastrointestinal symptoms, depression, anxiety, myalgia, arthralgia, migraine, tinnitus, and ear pain. The diagnosis of COVID-19-related POTS can be considered when such symptoms, particularly excessive increase in heart rate in the upright position, persist for more than 12 weeks after COVID-19 [13].

### 2.2. Presumed Mechanism for COVID-19-Related POTS

Beyond the severe respiratory effects observed in acute cases of COVID-19, the longer-term effects, known as long COVID, have attracted increased attention. Long COVID is estimated to affect at least 10% of subjects who had COVID-19, equating to approximately 65 million people globally, although this number is likely underestimated [27]. Long COVID is characterized by ongoing symptoms from 4 to 12 weeks after initial infection and post-COVID-19 syndrome, which occurs more than 12 weeks post-infection [13]. Long COVID can include a variety of conditions, particularly neurological disorders such as headache, vertigo, cognitive dysfunction, and autonomic dysfunction. Several pathophysiological theories have been suggested, such as immune system dysregulation. This dysregulation could aggravate immune responses and autoimmune reactions including reduced cardiovagal modulation and parasympathetic function impairment via molecular mimicry through viral infection [28]. To summarize, three presumed mechanisms have been proposed for long COVID.

#### 2.2.1. Renin–Angiotensin–Aldosterone System (RAAS) Dysregulation

Autoimmunity may be a probable, but main, process through which a coronavirus induces the creation of autoantibodies that target autonomic nerve fibers, as well as receptors for acetylcholine, adrenergic, and angiotensin II. Particularly, the angiotensin-converting enzyme (ACE) converts angiotensin I to angiotensin II, which results in vasoconstriction. Conversely, the ACE2 converts angiotensin I into angiotensin I-9 and angiotensin II into angiotensin I-7. Therefore, the ACE2 mainly acts opposite to the angiotensin II and plays a role in heart protection, including vasodilation, antioxidation, and anti-inflammatory actions [29]. In COVID-19, autoantibodies against ACE2, adrenergic receptors (ARs), and muscarinic acetylcholine receptors may be generated to provoke an autoimmune reaction [30,31,32,33,34]. The autoantibodies against ACE2 interfere with the normal function of ACE2, causing dysregulation of the RAAS.

#### 2.2.2. Hyperadrenergic Reaction

Autoantibodies to G-protein-coupled receptors were measured in a study of 31 patients with persistent long COVID-19 symptoms, and autoantibodies to β2-AR, α1-AR, angiotensin II AT1-receptor, nociceptin-like opioid receptor, and muscarinic M2-receptor were identified [33]. Increased AR antibodies due to COVID-19 infection may lead to vasoconstriction and tachycardia, which has been proposed as a possible mechanism in POTS [35].

#### 2.2.3. Direct Viral Infection

**Figure 2:**
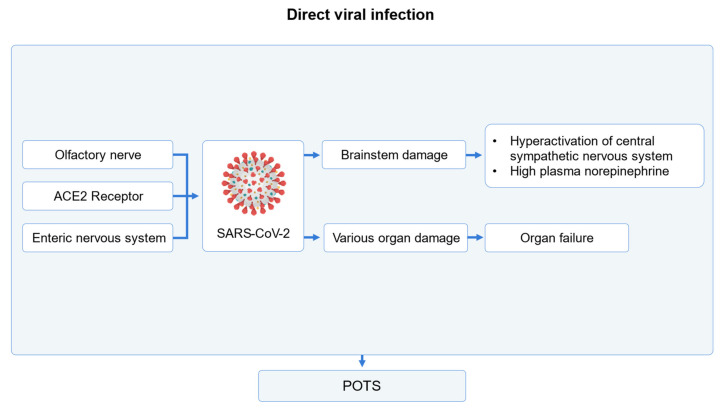
Illustration for brainstem and multi-organ damage via direct viral invasion in COVID-19-related postural tachycardia syndrome [36,37,38,39,40,41,42].

The COVID-19 virus can directly cause POTS symptoms (Figure 2). The COVID spike glycoprotein penetrates cells by attaching to ACE2 receptors, causing damage in multiple organs and RAAS dysregulation [36]. Similar to other viruses, COVID-19 can significantly influence the brainstem [37,38]. Because the brainstem plays an important role in the regulation of the cardiovascular system, autonomic nervous system, and neurotransmitter system [39], POTS symptoms due to injuries in the brainstem can occur. Furthermore, the COVID-19 virus can penetrate the human body through the olfactory nerve and may particularly damage central and autonomic nervous systems [40,41,42].

#### 2.2.4. Mechanism of Post-COVID-19 Tachycardia Syndrome

Post-COVID-19 tachycardia syndrome includes POTS as well as inappropriate sinus tachycardia. Such dysfunction in sinus tachycardia is caused by gain-of-function mutation of the pacemaker HCN4 channel [43], cardiac intrinsic sinoatrial nodule disorder, autoimmune system dysfunction, excessive sympathetic activation, or vagus nerve atrophy [44]. However, the mechanism for inappropriate sinus tachycardia after COVID-19 syndrome has not been clearly identified. Direct or indirect damage caused by COVID-19 viral infection can cause post-COVID-19 tachycardia syndrome. As mentioned above, the COVID-19 virus may cause multi-organ damage through ACE2 receptors. Damage may occur particularly in the lungs, kidneys, and heart and may trigger or aggravate arrhythmia and tachycardia [45,46,47,48]. Depletion of blood volume, neuroinflammation, anxiety and depression, pain, and fever caused by COVID-19 may also affect post-COVID-19 tachycardia syndrome [49,50].

## 3. Diagnosis of COVID-19-Related POTS

To date, accurate and validated diagnostic criteria for COVID-19-related POTS have not been established. Therefore, to diagnose COVID-19-related POTS, POTS first must be diagnosed. Subsequently, if POTS was caused or accompanied by COVID-19, COVID-19-related POTS should be considered.

Because various causes of tachycardia exist, diagnosing COVID-19-related POTS based on the presence of tachycardia is challenging. Many COVID-19-related POTS symptoms are based on postural changes, and the possibility of misdiagnosis cannot be ignored. Typical differential diagnoses include vasovagal syncope, orthostatic hypotension, dehydration, syncope, inappropriate sinus tachycardia, chromaffinoma, anxiety, depression, and unrecognized drug-related side effects [30]. Therefore, to diagnose COVID-19-related POTS, important components of diagnostic criteria for POTS should be considered. That is, in order to diagnose COVID-19-related POTS, the prerequisite that POTS occurred with or after COVID-19 infection must be met. It must also meet the general diagnostic criteria for POTS as follows. First, in an upright position, an increase of more than 30 beats per minute (more than 40 beats per minute for adolescents) without orthostatic hypotension might be suspected as POTS [11]. Unlike orthostatic hypotension, patients with POTS have unchanged or increased blood pressure in the upright position. Conversely, patients with POTS might show an increased heart rate for 30 to 60 s in the upright position [51]. This symptom normally is not observed in healthy subjects. Autonomic function tests, particularly the head-up tilt table test with electrocardiogram monitoring, are very useful for diagnosing POTS [52]. The head-up tilt test can be used to exclude orthostatic hypotension and check upright positional intolerance. During the head-up tilt test, the patient lies down on a table that is inclined gradually to as much as 70 degrees [52]. The heart rate, blood pressure, and oxygen concentration of the patient can be continuously monitored [52,53]. Furthermore, to diagnose COVID-19-related POTS, blood laboratory findings and electrophysiologic tests can be useful. Because symptoms associated with orthostatic tachycardia can occur in several diseases other than COVID-19-related POTS, other possible causes of orthostatic tachycardia should be excluded [31]. Blood laboratory examinations such as blood cell count, comprehensive metabolic panel, and thyroid function test can exclude non-cardiac causes of tachycardia such as hyperthyroidism and hypothyroidism. Electrocardiogram monitoring or holter monitoring can exclude symptoms associated with orthostatic tachycardia caused by underlying cardiac disease and a surge of cortisol due to circadian rhythm. Transthoracic echocardiography can exclude symptoms associated with orthostatic tachycardia caused by heart failure, abnormal plasma catecholamine levels, and electrolyte imbalance in blood and urine [52].

### Differential Diagnosis

For differential diagnosis of COVID-19-related POTS, two of the most important factors are history taking and physical examinations including vital signs and electrocardiogram in the upright position [11]. Therefore, differential diagnosis should be considered. In addition, a diagnosis of COVID-19-related POTS should be made after excluding all other possible diseases. Furthermore, COVID-19-related POTS and inappropriate sinus tachycardia share similar symptoms [11]. Inappropriate sinus tachycardia is defined as a heart rate greater than 100 beats per minute at rest and an average heart rate of more than 90 beats per minute over 24 h [44]. Inappropriate sinus tachycardia differs from COVID-19-related POTS because it occurs regardless of body position changes. Cardiac monitoring with an electrocardiogram can be used to distinguish between COVID-19-related POTS and inappropriate sinus tachycardia [44]. Generally, in POTS, a sudden positional change occurs when waking in the morning. Thus, cardiac monitoring in the morning is reportedly more sensitive for detecting POTS [54]. In orthostatic hypotension, blood pressure drops suddenly when the patient stands after sitting or lying down. This is evidenced by a sustained reduction in systolic blood pressure greater than or equal to 20 mmHg, or diastolic blood pressure greater than or equal to 10 mmHg within 3 min of standing, or head-up tilt to at least 60 degrees on a tilt table [55]. Whether the cause is neurotic or non-neurotic, heart rate changes appear secondarily to compensate for changes in blood pressure caused by body position changes [55]. However, in POTS patients, heart rate increases by more than 30 beats per minute compared to baseline, without the occurrence of orthostatic hypotension [52].

Most patients experiencing dehydration have an increased heart rate, which can be mistaken for COVID-19-related POTS [52]. Dehydrated patients may have accompanying hypovolemic conditions due to lack of body water and intravascular fluid as well as compensatory tachycardia. Thus, dehydrated patients usually present with orthostatic hypotension. Because orthostatic hypotension is not included in the diagnostic criteria for POTS, patients experiencing dehydration with compensatory tachycardia POTS can be excluded. In addition, certain drugs, such as thiazide and loop diuretic, promote body fluid loss and may lead to hypovolemia, which can also result in reflex tachycardia and hypotension [56]. Certain drugs can trigger tachycardia, which may complicate the diagnosis of COVID-19-related POTS [52]. Microstimulants, such as methamphetamine, cocaine, and caffeine, can increase heart rate through catecholamine mechanisms and by inhibiting phosphodiesterase [57]. In addition, commonly used bronchodilators such as albuterol can increase heart rate by activating β-Ars [57]. Antidepressants including fluoxetine and escitalopram can also increase heart rate via sodium and calcium inhibitory mechanisms [57]. Therefore, for proper diagnosis, underlying medication-related causes of COVID-19-related POTS-related symptoms must be identified. Most patients suffering from vasovagal syncope exhibit orthostatic symptoms such as sweating, dizziness, and nausea, similar to COVID-19-related POTS patients, only when they stand for long periods of time [58]. Presyncopal symptoms in POTS may resemble those in vasovagal syncope. However, in contrast to the immediate increase in heart rate during upright posture in POTS, there is a delayed, albeit abrupt, fall in blood pressure and heart rate with standing in vasovagal syncope. Increased heart rate or hypotension can be observed with psychological conditions such as anxiety and appears similar to COVID-19-related POTS symptoms [22]. Whether this is due to the high prevalence of anxiety in patients with COVID-19-related POTS remains unclear, and further research is needed.

## 4. Management

Reports of high-evidence randomized controlled trials on COVID-19-related POTS are scarce. Therefore, the management of COVID-19-related POTS is primarily based on existing guidelines or expert opinions. When treating COVID-19-related POTS, management should be tailored to the individual with a primary aim of alleviating symptoms to enhance the quality of life. The initial emphasis in treatment is on nonpharmacological therapies including patient education, lifestyle modification, and exercise [52,59]. However, pharmacological treatments may be necessary when COVID-19-related POTS-related symptoms are persistent or severe [9]. Next, general patient education and management methods based on the main mechanism of COVID-19-related POTS are reviewed.

### 4.1. Patient Education

An important aspect of COVID-19-related POTS management is patient education. Avoiding lying down, overeating, and high temperatures and humidity, and obtaining sufficient sleep should be primarily considered. These educational suggestions are presented in Table 1 [21,60,61,62,63,64].

### 4.2. Exercise

Appropriate exercise can increase the baroreflex sensitivity and reduce heart rate in the standing position. Gradually increasing exercise intensity can also relieve POTS-related symptoms and reduce abnormally increased heart rate in the standing position. Exercise intensity should be gradually increased from mild to moderate [59]. The standing position should be avoided at the outset, and the position during exercise should transition from semi-recumbent to upright. This exercise method may be particularly favorable for patients with COVID-19-related POTS [65]. Exercises for COVID-19-related POTS management should include both aerobic and anaerobic movements, such as thigh resistance training and muscular strength training [59].

### 4.3. Treatment for COVID-19-Related POTS Due to Hypovolemia

Regarding non-pharmacological intervention, a daily water intake of 3 L and a high-sodium diet (10 g daily) is recommended. Even in children, increasing appropriate salt intake for 1–3 months can be considered [14,58,64]. Sodium tablets should be avoided, which can cause gastrointestinal symptoms [16]. For patients who are unable to consume oral liquids, an intravenous bolus of 1–2 L normal saline can be used as a short-term strategy for up to 2 days [52]. For pharmacological intervention, fludrocortisone, an aldosterone analog, can temporarily increase intravascular volume and peripheral vascular resistance [12]. Because fludrocortisone can cause an increase in blood pressure, it is effective in treating low blood pressure. However, if COVID-19-related POTS patients have high blood pressure in the recumbent position, treatment with fludrocortisone can be dangerous. Furthermore, the use of fludrocortisone should be carefully considered because the drug can worsen hypokalemia. Desmopressin is a vasopressin analog that causes an increase in blood pressure by promoting water reabsorption [66]. Midodrine acts as a vasoconstrictor, reducing peripheral blood loading and improving orthostatic hypotension, and is especially recommended for the neuropathic subtype of POTS. The typical dose is 2.5 to 10 mg three times a day, administered during the day when the patient is awake and in an upright position [67]. Drugs that interfere with body fluid balance, such as diuretics, should be withdrawn in patients with POTS [21].

### 4.4. Treatment for COVID-19-Related POTS Due to Intravenous or Splanchnic Pooling

For non-pharmacological intervention, compression stockings of 30 to 40 mmHg can be used as well as abdominal binders of 10 mmHg in POTS [52]. Abdominal binders are more useful than compression stockings because significant amounts of venous blood are shifted from visceral veins via abdominal compression [68,69]. Regarding pharmacological intervention, midodrine can be used as mentioned above. In addition, droxidopa, a precursor of norepinephrine that may induce peripheral vasoconstriction, can be used for POTS management [70,71]. Octreotide, a somatostatin analog, can be prescribed for postprandial tachycardia and POTS symptoms because the drug reduces splanchnic pooling [72,73]. However, droxidopa and octreotide have not been commonly used as treatment for POTS and COVID-19-related POTS, so there is insufficient evidence.

### 4.5. Treatments for COVID-19-Related POTS Due to Increased Norepinephrine in the Blood

β-blockers can inhibit β-ARs that react to norepinephrine or epinephrine secreted from sympathetic nerve endings. The β-blockers help reduce tachycardia, decrease excess epinephrine, and alleviate orthostatic intolerance [52]. Propranolol is a non-selective β-blocker and is preferred over cardiac selective β-blockers to reduce pathogenic peripheral and visceral vasodilation [21]. Bisoprolol, metoprolol, and atenolol are β1-selective blockers. Such β-blockers are dangerous when used in patients with low blood pressure because they can lower blood pressure further. Ivabradine is a β-blocker analog that acts selectively in sinoatrial nodes, lowers heart rate during rest, and is effective in patients with low blood pressure. Ivabradine is typically injected twice a day at a dose of 2.5 to 20 mg to control the heart rate of POTS patients in the recumbent position to obtain a heart rate between 50 and 70 beats per minute. This improves the symptoms of upright tachycardia in POTS patients [74]. Clonidine and methyldopa may be effective for POTS by decreasing central sympathetic signals [75].

### 4.6. Treatment for COVID-19-Related POTS Due to an Autoimmune Mechanism

In COVID-19 cases, COVID-19-related POTS may also occur due to abnormalities in the immune response and autoimmune system, which can be managed with pyridostigmine, an acetylcholinesterase inhibitor that can be reversed. Pyridostigmine is typically administered in dosages ranging from 30 to 60 mg, three times daily. The drug helps reduce heart rate increase upon standing and alleviates the clinical symptoms of orthostatic intolerance in patients with POTS [76].

## 5. Conclusions

COVID-19 can cause a variety of tachycardias, including inappropriate sinus tachycardia as well as POTS. However, due to nonspecific symptoms of POTS, distinguishing COVID-19-related POTS from other diseases can be difficult. Thus, specific differential diagnosis of COVID-19-related POTS should be conducted for COVID-19 patients. Patients with COVID-19-related POTS may need to modify their lifestyle based on non-pharmacological treatment recommendations. Pharmacological treatment based on the individual situation of COVID-19-related POTS patients can also be administered.

## Figures and Tables

**Table 1:** Non-pharmacological and pharmacological treatments based on causes of POTS associated with COVID-19.

	Common	Hypovolemia	Intravenous/Splanchnic Pooling	Blood Norepinephrine Level Increase	Autoimmunity
Non-pharmacological treatment	Avoid lying down for a long time. Stand up gradually, especially after meals and urination. Avoid exposure to high temperature and humidity. Avoid eating a large amount at once. Physical counter-pressure maneuvering and contraction of gastrocnemius muscle and tensor muscle. Maintain good sleep hygiene. Exercises: aerobic exercise and thigh resistance training.	10 g or more of sodium per day/water 3 L or more. Elevating head-end of the bed 10 cm for sleeping. Sleeping in seated position.	Compression stockings, clothes. Eating habits: consuming small amounts of food multiple times per day or conducting food elimination.		
Pharmacological treatment		Fludrocortisone Desmopressin Midodrine Withdrawal of drugs that interfere with liquid balance	Midodrine Droxidopa Octreotide	Propranolol Bisoprolol Metoprolol Atenolol Ivabradine Clonidine Methyldopa	Pyridostigmine
